# Decreased Response to Acetylcholine during Aging of *Aplysia* Neuron R15 

**DOI:** 10.1371/journal.pone.0084793

**Published:** 2013-12-27

**Authors:** Komolitdin Akhmedov, Valerio Rizzo, Beena M. Kadakkuzha, Christopher J. Carter, Neil S. Magoski, Thomas R. Capo, Sathyanarayanan V. Puthanveettil

**Affiliations:** 1 Department of Neuroscience, The Scripps Research Institute, Scripps Florida, Jupiter, Florida, United States of America; 2 Department of Biomedical and Molecular Sciences, Queen's University, Kingston, Ontario, Canada; 3 Division of Marine Biology and Fisheries, University of Miami Rosenstiel School of Marine and Atmospheric Science, Miami, Florida, United States of America; Texas A&M University - Corpus Christi, United States of America

## Abstract

How aging affects the communication between neurons is poorly understood. To address this question, we have studied the electrophysiological properties of identified neuron R15 of the marine mollusk *Aplysia californica*. R15 is a bursting neuron in the abdominal ganglia of the central nervous system and is implicated in reproduction, water balance, and heart function. Exposure to acetylcholine (ACh) causes an increase in R15 burst firing. Whole-cell recordings of R15 in the intact ganglia dissected from mature and old *Aplysia* showed specific changes in burst firing and properties of action potentials induced by ACh. We found that while there were no significant changes in resting membrane potential and latency in response to ACh, the burst number and burst duration is altered during aging. The action potential waveform analysis showed that unlike mature neurons, the duration of depolarization and the repolarization amplitude and duration did not change in old neurons in response to ACh. Furthermore, single neuron quantitative analysis of acetylcholine receptors (AChRs) suggested alteration of expression of specific AChRs in R15 neurons during aging. These results suggest a defect in cholinergic transmission during aging of the R15 neuron.

## Introduction

 Normal aging is characterized by progressive deterioration of several functions [1-3]. What is the neurophysiological basis for the decline in brain function during normal aging? Previous studies have identified alterations in synapse number and synapse physiology during the aging process. In older mice, dendritic spines are smaller. This results in weaker synapses, which then form less efficient circuits [4]. Brain aging is also characterized by the loss of synapses. For example, electrophysiological and anatomical studies of the CA1 and dentate gyrus of hippocampus in older rats have shown significant loss of synapses, deficits in induction and maintenance of long-term potentiation (LTP), lower thresholds for long-term depression (LTD) and depotentiation [3,5-7]. The deficit in hippocampal LTP affects its communication with other structures. Almaguer et al showed that aging impairs communication between the amygdala and hippocampus in older rats [8]. 

 Aging also differentially alters components of neural circuitry. In a study that measured long-term potentiation (LTP) in middle aged and young rats, Rex et al showed that LTP at basal dendrites induced by theta burst stimulation decayed rapidly in older rats, whereas LTP induced at apical dendrites did not show any significant difference [11].

 Aging impacts brain regions differentially. For example, in rats, the cerebellum showed earlier senescence in the hippocampus. Stereological studies of rats at different ages have shown significant loss of cerebellar Purkinje neurons, whereas hippocampal pyramidal neurons were stable across age groups [9]. Moreover, there were significant deficits in cerebellar long-term depression (LTD) in older rats. In monkeys, the frontal and temporal cortex showed differential aging. The synaptic numeric density (number of synapses per micrometer of the tissue) significantly decreased in the temporal cortex, whereas in the frontal cortex, there was no significant change during aging [10]. 

 Despite these studies, we still know little about how aging affect electrophysiological properties of defined circuits and identified neurons that underlie specific behaviors. Such knowledge will advance our understanding of how aging alters specific properties of components of neural circuitries and the molecular substrates of aging. To address this challenge we used the well-defined circuits and identified neurons of the marine snail *Aplysia*. Neurons of *Aplysia* are larger and easily tractable from animal to animal [12], facilitating single neuron analysis of aging. 

*Aplysia* has been used to study age-dependent changes in anatomy and function in identified neurons [13-16] behavioral changes [17,18], expression of mRNA [19], neurotransmitter regulation [20-22], and circadian rhythms [23]. Recently, effects of temperature on aging *Aplysia* [24], changes in D-aspartate ion currents [25], and gene expression in identified neurons R2 and Lpl2 [26] during aging were described. 

**Figure 1:**
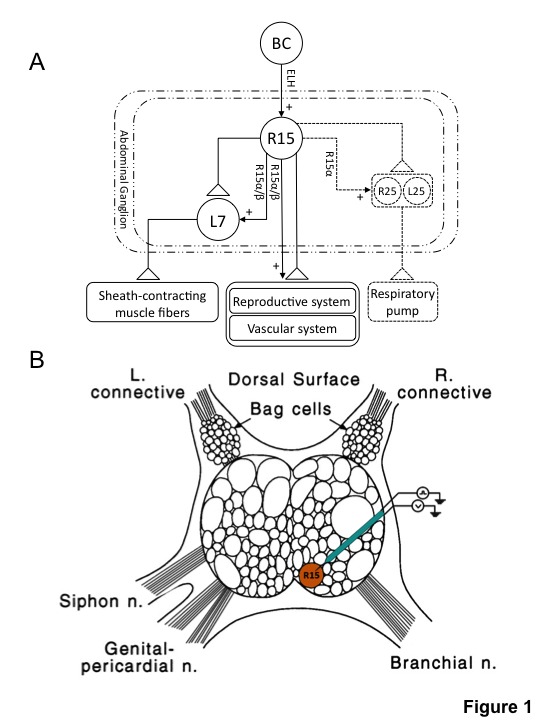
R15 neuron of *Aplysia*. (**A**): Schematic diagram of the known synaptic connections made by R15 in the abdominal ganglion and the behaviors in which R15 has a potential role. L7, R25 and L25 are three known neurons to which R15 interacts with. R15 α and β are peptides produced by R15 neuron (modified from Alveizos et al., 1992). Triangles represent chemical excitatory connections. Arrows with “+” sign indicate positive regulation. BC: bag cells; ELH: egg laying hormone (B): Recording from R15 neuron. Location of R15 in the abdominal ganglion is shown in the cartoon. L: left, R: right, n: nerve.

 Here, we report specific electrophysiological changes in neuron R15 during aging. R15 is a bursting neuron in the abdominal ganglia [27-30] and participates in reproduction, circulation, respiration, and water balance [31-33]. Neurons that interact with R15 and its implicated physiological functions are shown in Figure 1. To understand the effect on aging on electrophysiological properties, we studied neurotransmitter acetylcholine-induced changes in rhythmic bursting activity of the R15 neuron using a methodology we recently described [34]. We find that the R15 neuron from mature and old animals did not show significant changes in the membrane resting potential and latency in response to acetylcholine, however, the burst number and burst duration are altered during aging. Specific defects during aging in different components of action potential waveform, such as amplitude and duration of depolarization and repolarization, were identified. Furthermore, we find that aging alters expression of specific nicotinic acetylcholine receptors in the R15 neuron. 

## Results

 In laboratory conditions (12-15 °C and well fed), *Aplysia* reaches sexual maturity in 5-6 months and live up to 12-14 months [24,35]. However, this duration can be prolonged depending on temperature and availability of food [24]. To study how aging affects the electrophysiological properties of neurons, we have raised a cohort of 34 animals at the National Facility for Aplysia at the University of Miami Rosenstiel School of Medicine and carried out intracellular recordings of the R15 neuron from *Aplysia* at two different growth stages (6-7 months and 11-12 months old). Using a modified methodology for carrying out intracellular recordings from abdominal ganglia [34], we recorded membrane potentials and bursting action potentials from R15 neurons of mature and old animals (Figure 1). 

### Resting membrane potentials do not change in older animals

**Figure 2:**
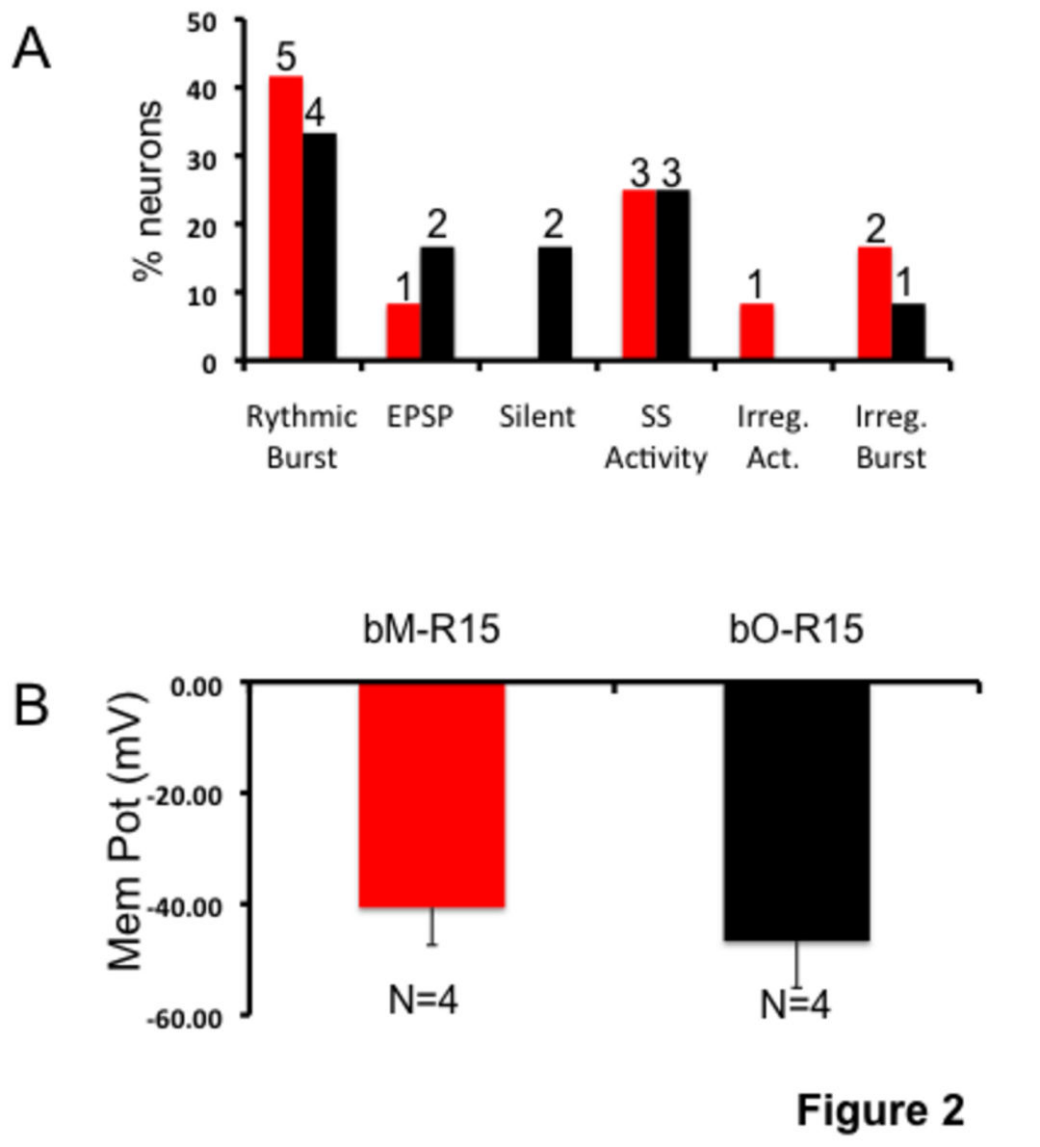
Electrophysiological measurements in R15 neuron in the intact ganglia. A: Six types of bioelectric activity were recorded in R15 neurons. Percent R15 neurons with each activity are shown in bar graphs. Red bars: Mature; Black bars: Old. Numbers of neurons that showed specific activity is shown on each bar graph. B: Resting membrane potential of rhythmic bursting R15 from mature (bM-R15) and old (bO-R15) animals. Red: bM-R15 and black: bO-R15. Statistical analysis suggests that there are no significant differences (n=4; Student’s t test, p > 0.05) in resting membrane potentials of bM-R15 and bO-R15 neurons.

 To study how aging affects electrophysiological properties of the R15 neuron, we first measured the resting membrane potentials. Bioelectric activity and discharge patterns allowed us to discriminate six different R15 firing patterns (Figure 2A; Table S1 in File S1) in R15 neurons from Old (O-R15; n = 12) and Mature (M-R15; n = 12) animals: rhythmic burst firing (M-R15 = 42%; O-R15 = 33%), EPSP (M-R15 = 8%; O-R15 = 17%), silent (M-R15 = 0%; O-R15 = 17%), single spike (M-R15 = 25%; O-R15 = 25%), irregular activity (M-R15 = 8%; O-R15 = 0%) and irregular burst firing (M-R15 = 17%; O-R15 = 8%). χ^2^ test showed no significant difference between various R15 activities in old and mature R15 neurons. 

 In our subsequent measurements and analyses, we focused only on R15 neurons that showed rhythmic bursting activity, hereafter respectively named bM-R15 and bO-R15 for rhythmically bursting mature and old R15 neurons. bO-R15 showed a mean membrane resting potential of -46.70 ± 14.54 mV (n=4); by contrast, the bM-R15 mean membrane resting potential was -40.65 ± 11.75 mV (n=4). The statistical comparison of the mean membrane resting potential of bM-R15 vs. bO-R15 neurons did not show any significant difference (Student’s t test, p = 0.54, F: 0.42, DF: 7; Figure 2B; Table S2 in File S1). Mean membrane potential analysis of all the 12 neurons (R15 from old and mature animals) also did not show any statistical significant difference (Student’s t test, p > 0.05, DF: 23; Figure S1). 

### Aging does not change the response latency and shift in depolarization

**Figure 3:**
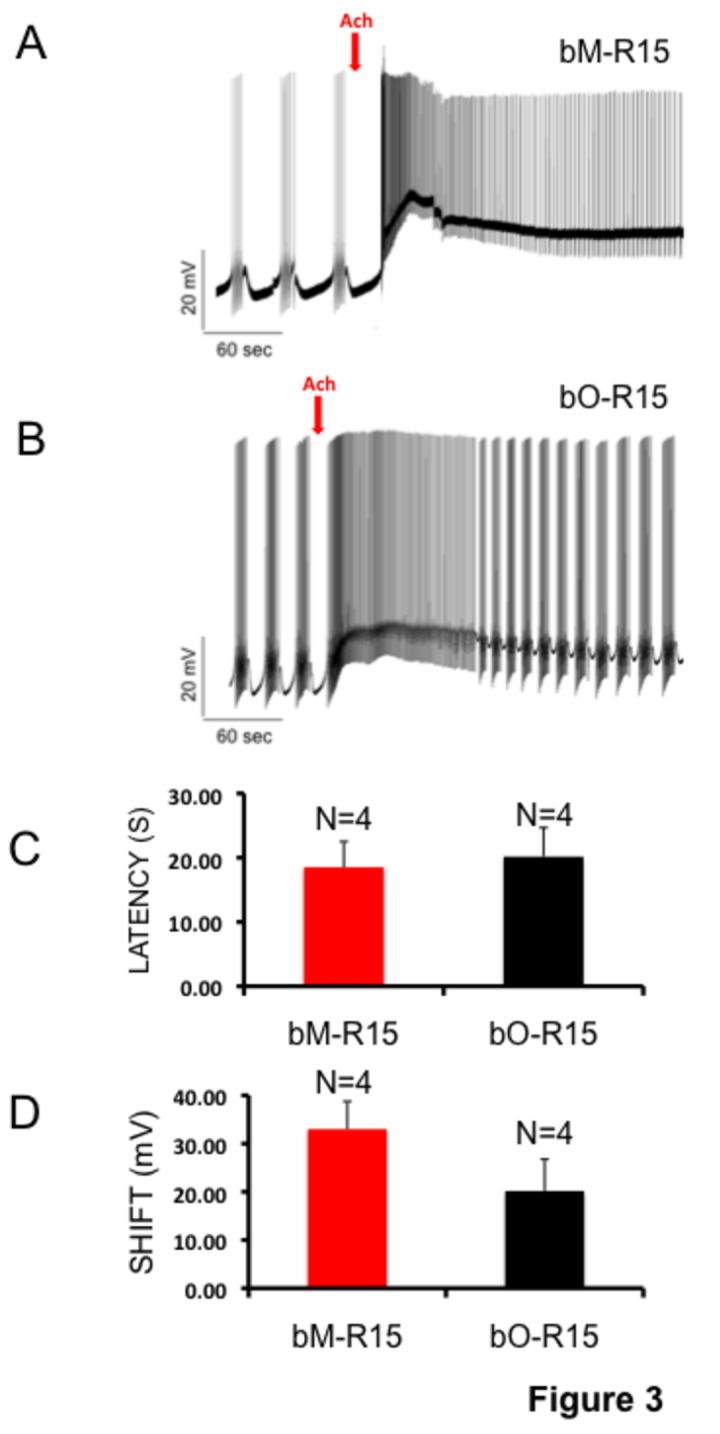
Rhythmic bursting activity of R15. The R15 bursting pattern activity was recorded for 30 minutes or more in order to continuously check the presence of stable rhythmic activity before drug application. Typically, bursts are characterized by groups of 2–20 spikes, separated by long (2–30 second) post burst hyperpolarizations. Representative burst raw trace before and after acetylcholine (ACh) application from bM-R15 (A) and bO-R15 (B) neurons is shown. The red arrow indicates the point of acetylcholine application. Calibrations are indicated in the figure. In both bO-R15 and bM-R15 group 1 mM ACh exposure elicited an increase in bursting. On washout (~2 minutes after ACh application), the effect was fully reversed. The recordings were amplified using a DC pre-amplifier. C: Bar graph showing the mean latency of effect after 1 mM ACh application in R15 bursting neurons. D: Bar graph showing the mean shift in depolarization after 1 mM ACh application in R15 bursting neurons. There are no significant differences (n=4; Student’s t test, p > 0.05) between bM-R15 and bO-R15 neurons. Red bars: bM-R15 neurons; Black bars: bO-R15 neurons. Error bars are SEM.

 We assumed that if aging affects neural circuit function, there must be changes in response to specific neurotransmitters during aging. We focused on the response to the neurotransmitter acetylcholine (ACh) because previously it was shown that R15 respond to acetylcholine exposure [70,71], and several studies have shown that the cholinergic system is affected during aging [36-43]. Bursting activities were recorded from the bM-R15 neuron and bO-R15 before and after ACh application (Figures 3A & B; Figure S2). ACh (final concentration 1 mM) was applied by micropipetting into the recording chamber that was continuously perfused with ASW. bO-R15 neurons responded to ACh with an average latency of 18.51 ± 6.93 seconds and a mean shift in depolarization from baseline of 33.00 ± 9.97 mV (Figure 3C & D). bM-R15 neurons responded to ACh application with a latency of 20.16 ± 7.82 seconds and a mean shift in depolarization from a baseline of 20.18 ± 11.37 mV (Table S2 File S1; Figure 3C & D). Statistical analysis of the data did not show any significant difference in latency (Student’s t test, p = 0.76, F: 0.1, DF: 7) or shift amplitude (Student’s t test, p = 0.14, F: 2.88, DF: 7) between the two groups, suggesting that aging did not change the initial response to ACh.

### Burst number and duration change during aging

 We next studied aging-associated changes in burst firing by measuring the number of action potentials in a burst and duration of burst firing before and after ACh exposure. We studied changes in burst firing first by measuring the average number of action potentials in each burst and duration of bursts, considering a window time of 90 seconds, before and after ACh exposure (Figure S2; Table S3 File S1). These measurements were analyzed by One-Way ANOVA and Tukey’s HSD post hoc test to identify statistically significant differences.

**Figure 4:**
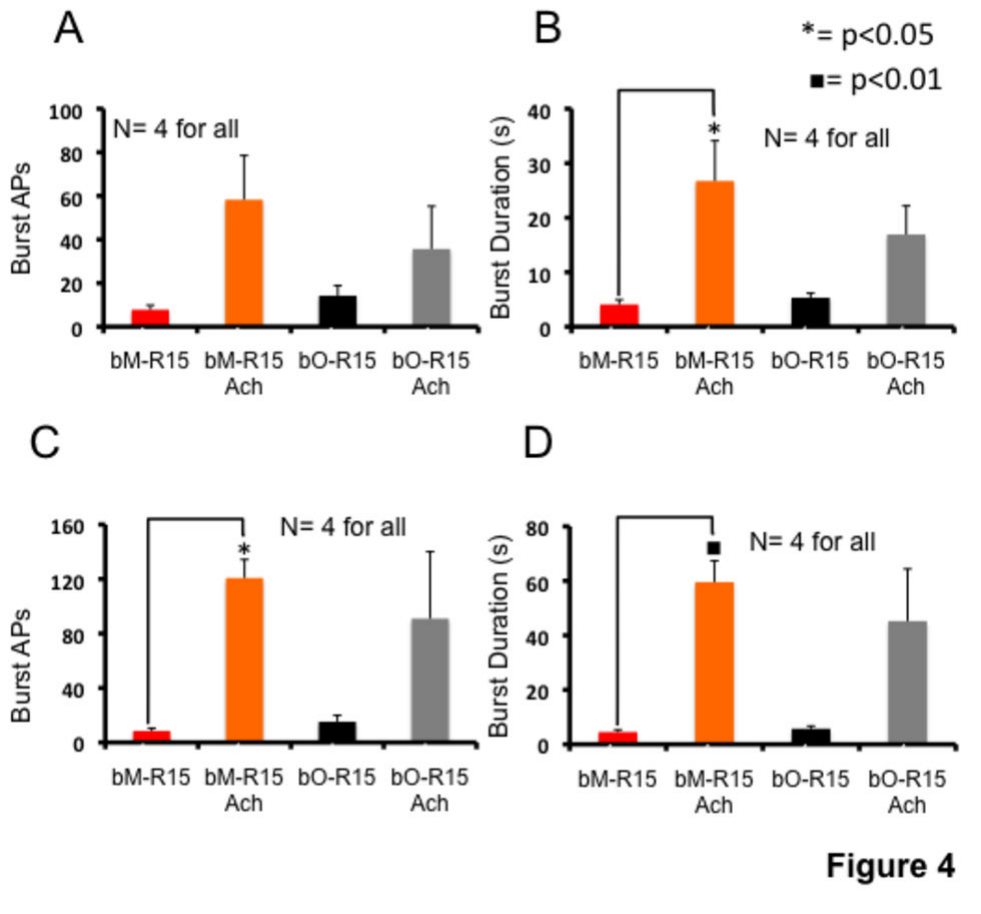
Analysis of burst firing of mature and old R15 neurons. Histograms showing the average number of APs (A) and average duration (B), of R15 bursts recorded from bO-R15 and bM-R15 neurons 90 seconds before and after application of 1 mM ACh. C and D: analysis of number of APs (C) and duration (D) considering only the first burst after ACh application. Values are expressed as mean ± SEM. s: seconds; an asterisk or a black-filled square indicates statistically significant differences. Differences were considered significant (n=4; One way ANOVA and Tukey’s HSD test) at the level of p < 0.05 (asterisk) or p < 0.01 (black filled square). bM-R15: rhythmic bursting R15 from mature animals; bO-R15: rhythmic bursting R15 from old animals, bM-R15 ACh and bO-R15 ACh are bM-R15 or bO-R15 neurons after ACh exposure.

 We found that in bO-R15 neurons, before ACh application, the average number of action potentials (APs) per burst was 14.24 ± 7.93 (Figure 4A) and the average duration was 4.6 ± 1.9 sec (Figure 4B). ACh application increased both burst average number of APs (35.67 ± 33.98) and the average burst duration (13.23 ± 10.47 sec) of bO-R15 neurons, but the changes were not statistically significant (p = 0.25, F: 3.41 for number of APs and p = 0.49, F: 6.43 for burst duration; DF: 15). The average number of APs per burst or duration of bursting in bM-R15 and bO-R15 were not significantly different (average number of APs per burst in bM-R15: 7.80 ± 3.54; p = 0.98, F: 3.41, DF: 15; Figure 4A; average duration of bursting in bM-R15: 4.09 ± 1.50 sec; p = 1.00, F: 6.43, DF: 15; Figure 4B). We find that that ACh induced changes in the average number of APs in bursting were not significant in bM-R15 neurons (58.30 ± 35.04; p = 0.06, F: 3.41, DF: 15), however changes in the burst duration was significant (26.74 ± 12.80, p < 0.05, F: 6.43, DF: 15). 

 We then analyzed the first burst following 1 mM ACh exposure. ACh-induced changes in the number of APs and duration of the first burst are significant in bM-R15 (number of APs: p < 0.05, DF: 15, F: 6.32; duration: p < 0.01, DF: 15, F: 9.54) whereas not significant in bO-R15 (number of APs: p = 0.13, F: 6.32, DF: 15; duration: p = 0.09, F: 9.54, DF: 15; Figures 4C and D). 

### Analysis of R15 action potentials in mature and old neurons

**Figure 5:**
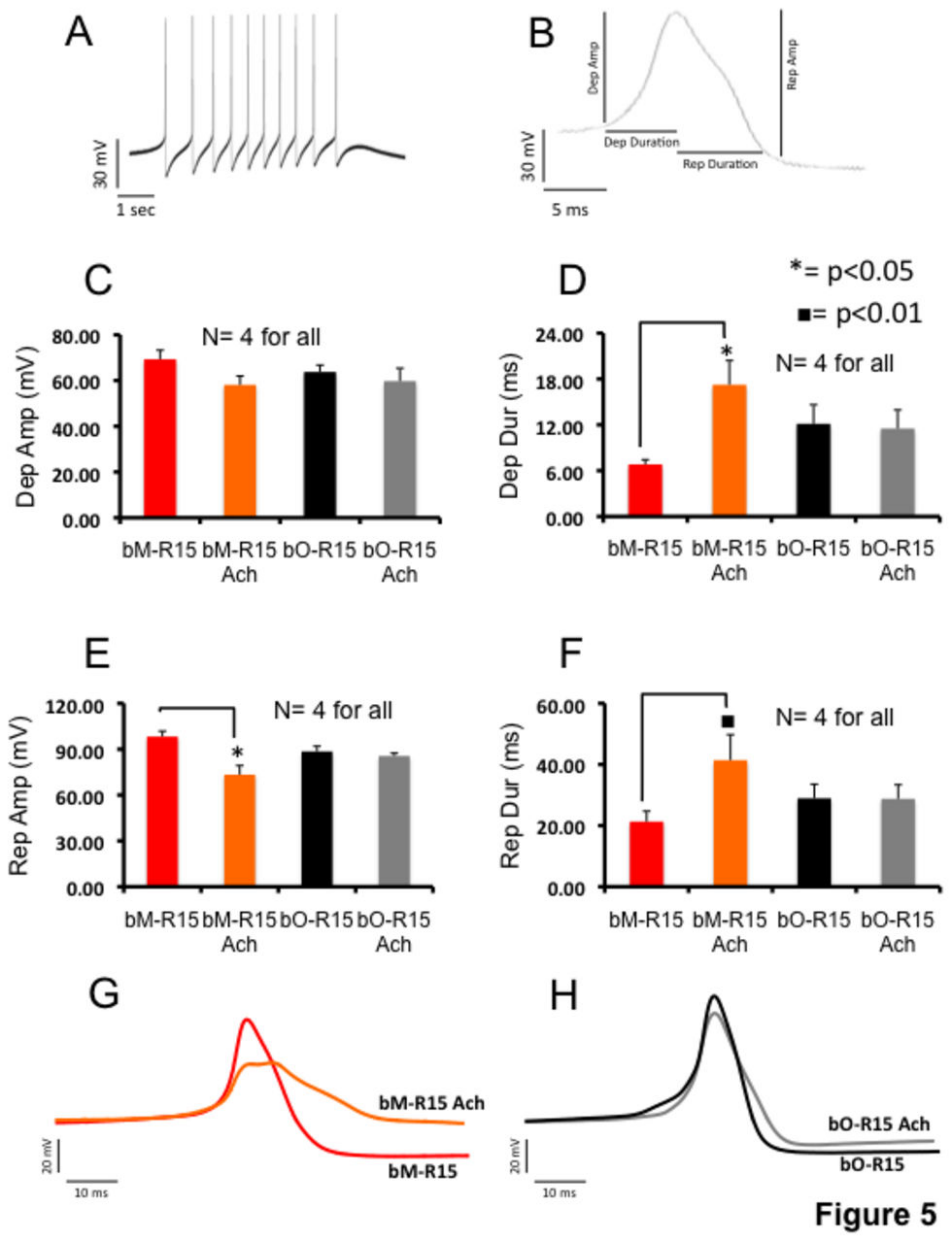
Analysis of action potential waveform in rhythmic bursting R15 from mature and old animals. A: action potentials in a single burst. B: measurements of waveform parameters (Depolarization amplitude, Depolarization Duration, Repolarization amplitude, Repolarization duration). Bar graphs showing the mean depolarization amplitude (C), the mean depolarization duration (D), repolarization amplitude (E), repolarization duration (F) of AP waveforms recorded in rhythmic R15 bursting neurons from old and mature animals, before and after 1 mM ACh application. Values are expressed as mean ± SEM. mV: milli volt; msec: milli seconds. An asterisk or a black-filled square indicates statistically significant differences. Differences were considered significant (n=4; One way ANOVA and Tukey’s HSD test) at the level of p <0.05 (asterisk) or p <0.01 (square). G & H: Representative action potentials waveforms of R15 bursting activity recorded in bM-R15 (G) and bO-R15 (H) neurons before and after 1 mM ACh application. bM-R15: rhythmic bursting R15 from mature animals; bO-R15: rhythmic bursting R15 from old animals, bM-R15 ACh and bO-R15 ACh are bM-R15 or bO-R15 neurons after ACh exposure.

 Next, we analyzed different parameters of action potential (AP) waveform, such as amplitude and duration of depolarization and repolarization of bM-R15 and bO-R15 bursts (Table S4 in File S1; Figures 5A & B). Off-line analysis of the abovementioned four different parameters of burst AP waveform before and after ACh application identified specific changes in AP waveform during aging. One-way ANOVA and Tukey’s HSD post hoc test were used to identify statistically significant differences in different AP waveform parameters (Table S4 in File S1). Before ACh application, bO-R15 APs had mean depolarization amplitude of 63.77 ± 5.19 mV (Figure 5C) with depolarization duration of 12.14 ± 4.32 msec (Figure 5D), a mean repolarization amplitude of 88.54 ± 5.93 mV (Figure 5E) with a repolarization duration of 28.94 ± 8.00 msec (Figure 5F). Similarly, bM-R15 APs had a mean depolarization amplitude of 69.34 ± 6.81 mV, and depolarization duration of 6.82 ± 1.05 msec, mean repolarization amplitude of 98.28 ± 6.14 mV and mean repolarization duration of 21.31 ± 5.90 msec (Figures 5E and F). Statistical analysis between the bM-R15 vs. bO-R15 group before ACh application showed no significant difference for depolarization amplitude (p = 0.69, F: 1.87, DF: 15), depolarization duration (p = 0.31, F: 4.32, DF: 15), repolarization duration (p = 0.68, F: 2.99, DF: 15) and amplitude (p = 0.24, F: 8.97, DF: 15).

 After ACh application, bO-R15 burst APs had a mean depolarization amplitude of 59.76 ± 9.77 mV, with a duration of 11.52 ± 4.21 msecs, mean repolarization amplitude of 85.59 ± 3.15 mV with a duration of 28.77 ± 7.95 msec (Figures 5C,D, E & F). bO-R15 burst APs waveform parameters before and after ACh application did not show any statistically significant difference (depolarization amplitude: p = 0.9, F: 1.88, duration: p = 0.31, F: 4.32 and repolarization duration: p = 0. 7, F: 3; amplitude: p = 0.24, F: 8.97; DF: 15). 

 After ACh application, bM-R15 bursts APs, had a mean depolarization amplitude of 58.11 ± 6.65 mV, with a duration of 17.25 ± 5.47 msec, and a mean repolarization amplitude of 73.38 ± 10.25 mV, with a duration of 41.41 ± 14.48 msec (Figures 5C and D). Statistical analysis of data showed a significant increase of both depolarization (p < 0.05, F: 4.32, DF: 15,) and repolarization duration (p = 0.05, F: 3, DF: 15), and at the same time, a significant decrease of repolarization amplitude (p < 0.01, F: 8.97, DF: 15) due to ACh exposure. Statistical comparison of bM-R15 and bO-R15 neurons after ACh application did not show any significant differences in repolarization amplitude (p = 0.11, F: 8.97, DF: 15). Representative wave forms of burst APs from bM-R15 and bO-R15 neurons (Figure 5G & H) show specific changes in response to ACh. 

### Quantitative analysis of expression of ACh receptor genes in R15 during aging

 The finding that bO-R15 does not respond well to ACh led us to consider the possibility that aging is associated with a decrease in the expression of ACh receptors in R15. The decrease in the availability of ACh receptors (AChRs) might cause the poor response to ACh as the animal ages. To test this possibility, we quantified the expression of known ACh receptors in *Aplysia*. We obtained 8 nicotinic AChR sequences from NCBI (www.ncbi.nlm.nih.gov/). *Aplysia* homologs for muscarinic AChR sequences were not found in the current database. We first analyzed the expression of nicotinic AChRs in abdominal ganglia (data not shown), and then in single R15 neurons. We found that 5 out of the 8 AChRs we studied are expressed in the R15 neurons. Comparisons of protein sequences of these five AChRs (AChR-C, AChR-E, AChR-G, AChR-J1 and AChR-Q) suggested that these genes code for unique nicotinic AChRs (Figure 3). 

**Figure 6:**
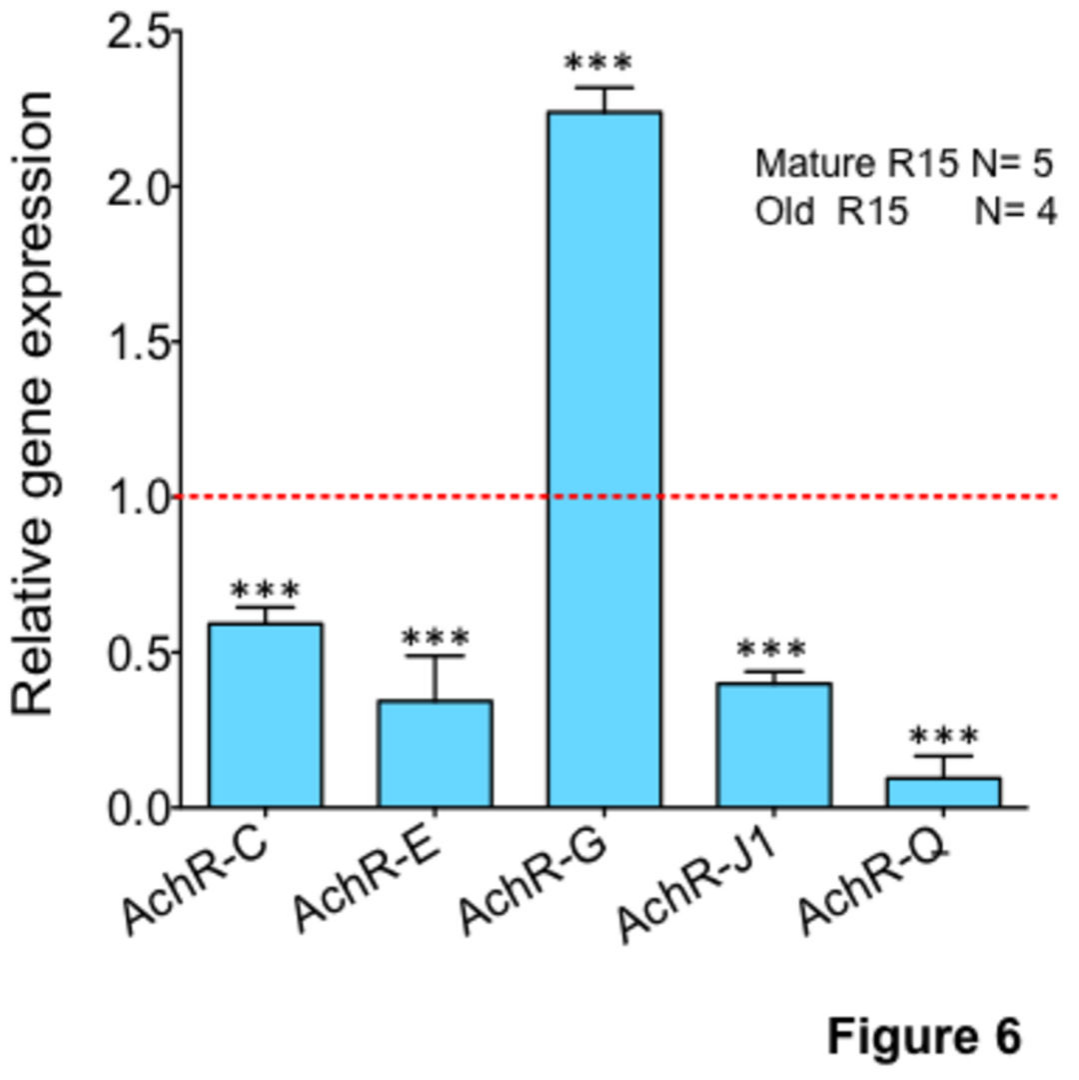
Quantitative analysis of AChRs in single R15 neurons from mature and old animals. RNAs isolated from single R15 neurons were amplified and used for qPCR analysis. Data was normalized to 18S rRNA levels. Relative expression of five AChRs in old R15 neurons is shown in bar graphs. Fold change ± SEM are shown. One way ANOVA and Tukey’s HSD post hoc test was used to determine the statistical significance where **p value < 0.01, ***p value < 0.001.

 We next quantified the expression of these 5 AChRs by quantitative PCR (qPCR) analysis of RNAs prepared from single R15 neurons isolated from old and mature animals (Tables S5 and S6 in File S1). The relative expression of AChRs in old neurons is shown in Figure 6. Data was first normalized to 18S rRNA levels. We find that expression of AChR-C, AChR-E, AChR-J1 and AChR-Q showed a significant decrease in expression in old animal R15 neurons (fold decrease in expression: AChR-C, 2 ± 0.6; AChR-E, 2 ± 0.4; AChR-J1, 2 ± 0.4; AChR-Q, 10 ± 0.1, n=5 for mature and n=4 for old; p < 0.0001; One way ANOVA and Tukey’s HSD multiple comparison test; Table S6 in File S1), whereas AChR-G showed a significant increase in expression (fold increase: 2 ± 0.2, n=5 for mature and n=4 for old, p < 0.0001; One way ANOVA and Tukey’s HSD post hoc test; Table S6 in File S1).

## Discussion

 Aging research has several distinctive challenges. Because of the long life span of humans and various ethical issues, it is difficult to carry out aging studies in humans. Several species of animals have been used as animal models to study aging. Much of these studies have focused on the genetic basis of aging and understanding physiological pathways that regulate the rate of aging and age related diseases. In general, aging is associated with a decline in function and a greater risk of diseases. For example, it is established that aging is associated with a progressive decline in our cognitive abilities, such as learning and memory, vision, hearing, and motor skills. Decades of research have identified several features of an aging brain. These include specific physiological, molecular, and cellular changes in the brain. However, it is not yet well understood how a specific circuitry or neuron causes specific behavioral changes during aging. In this study, we explored the advantages of *Aplysia* and examined age dependent changes in basal electrophysiological properties, response to acetylcholine and quantified mRNA expression levels of multiple acetylcholine receptors in single neurons. Specifically, we characterized age-dependent changes in the electrophysiological properties of the identified neuron R15. The R15 neuron is of interest because of its role in *Aplysia* behaviors, such as egg laying, osmoregulation and cardiac function, and its characteristic bursting activity.

 We studied the R15 neuron in sexually mature adults (~6 months from hatching) and older (~12 months from hatching) animals. Considering that *Aplysia* live up to 12-14 months in the laboratory, the older animals we studied are close to senescence. Our electrophysiological measurements of mature and old R15 neurons from the cohort of 34 animals suggested that these neurons show six different properties. Briefly they exhibited rhythmic bursting, irregular bursting, EPSPs, single spike, irregular activity and silent activity. Statistical analysis of this data did not find any significant differences in percentage of neurons that show different activities between mature and old R15 neurons. We do not yet understand the physiological significance of these activities. Because regular bursting activity is a well-studied characteristic of R15 [27-33], for the subsequent analysis we only focused on R15 neurons that showed rhythmic bursting. 

 We first assumed that aging negatively affects synaptic transmission, and that membrane properties of R15 are altered during aging. Contrary to this idea, we found that membrane potentials of R15 did not significantly change in older animals. Next, we studied the latency and shift in depolarization of ACh-induced changes in bursting. The latency and shift in depolarization are readouts for how fast a neuron is able to respond to a neurotransmitter. R15 is an Ach responsive neuron, and we expected a difference in latency and shift in depolarization in the R15 neurons of old animals. Interestingly, our analyses show that these parameters do not change significantly during aging, suggesting that regular bursting R15 neurons of old animals (bO-R15) are able to respond to ACh. Though there was a trend for the shift in depolarization to change in old R15 neurons, the change did not reach statistical significance in our study. Consistent with these observations on latency in shift in depolarization, Cepeda et al have shown that basic electrophysiological parameters such as resting membrane potential did not change during aging of the neostriatal neurons of rats [44].

 Next, we analyzed two parameters of bursting, the number of action potentials in a burst, and duration of bursting. We did this in two ways: first, by analyzing measurements over the period of 90 seconds, and second, by studying the first burst in response to ACh. We found that the bO-R15 neurons burst slightly more than bM-R15 neurons, but without reaching the level of statistical significance. ACh did not increase the average number of action potentials of bM-R15 bursts. However, a significant increase of the mean burst duration was observed in response to ACh. More importantly, when the first burst following ACh exposure was analyzed, ACh induced significant changes both in the number of APs and the duration of burst of bM-R15 neurons. By contrast, ACh did not change either of these parameters of bO-R15 bursts. These measurements suggest that, though the old R15 is able to initiate a response to ACh, this response was not sustained and translated into changes in the number of action potentials and duration of bursting. Interestingly, the bO-R15 bursts had a higher number of APs per burst, however the increase did not reach the level of statistical significance.

 Bursting activity can be intrinsic or input driven, as in the case of ACh-induced changes in R15 bursting. Bursting is a complex neuronal firing pattern and is regulated by complex feedback mechanisms [27,45-47]. Input driven bursting of R15 should be controlled by ACh released onto R15 by the presynaptic neurons. Few neurons that are presynaptic to R15 are described. For example L10 neuron in the abdominal ganglia [70] is presynaptc to R15. Tremblay et al [72] has shown that stimulation of the right connective nerve cause release of acetylcholine to R15. We mimicked an ACh release by a brief application of ACh into the bath. Our measurements of bursting in old and mature neurons suggest that the machinery that control ACh-induced changes in bursting become defective in the R15 neuron as the animal ages. Thus, these results suggest specific changes in cholinergic transmission during aging.

 To gain deeper insight into the changes in ACh-induced bursting during aging, we analyzed burst action potential waveforms. We studied four different parameters of action potential waveforms, such as the amplitude and duration of depolarization and repolarization [48]. We considered two possibilities: (1) During aging, AP waveform parameters are altered in rhythmic bursting R15 neurons and (2) ACh induced changes in AP waveform parameters are altered during aging. The first possibility would inform us whether there are differences in voltage-gated sodium and potassium channels that regulate AP generation during aging, and the second possibility will test whether there is specific defect in burst APs produced by bO-R15 neurons in response to ACh. 

 Our analysis suggests that there are no significant differences in depolarization amplitude of burst APs before or after ACh exposure to bM-R15 and bO-R15 neurons, suggesting that the rising phase of action potential (sodium influx) do not change during aging. However, ACh exposure increased the duration of AP in bM-R15 neurons, whereas it did not affect the duration in bO-R15 neurons. These results suggest that there are changes in the membrane excitability of R15 as *Aplysia* age, which is consistent with the findings in the mammalian brain, that aging is associated with a change in membrane excitability [49-53]. 

 As in the case of depolarization amplitude, the repolarization amplitude did not change suggesting normal resetting of voltage-gated sodium channels and opening of voltage-gated K channels [48] in both bM-R15 and bO-R15 neurons. However, ACh exposure, unlike in bO-R15 neurons, caused a decrease in the repolarization amplitude and an increase of duration in bM-R15 neurons, suggesting that resetting of voltage-gated sodium channels and opening of potassium channels are altered. Consistent with these observations, several studies report aging-associated changes in properties of ion channels, such as potassium channels [54-56], calcium channels [52,57,58], and sodium channels [59]. Thus, analysis of AP waveforms support the second possibility that Ach induced changes in waveform parameters are altered in old R15 neurons. Taken together, both the analysis of bursting and AP waveform parameters, our results suggest specific defects in ACh responsiveness during aging of R15.

 We next analyzed Ach responsiveness by quantifying the expression of AChR genes in R15. ACh upon binding to ACh receptors (AChRs) cause an increased flow of positive charge, including the flow of sodium and potassium ions increasing a net positive charge in neurons. This net increase in sodium and potassium ions (due to activation of AChRs) causes changes in the shape of action potential. Our data suggest that AChRs are defective in bO-R15 neurons such that ACh binding does not lead to an increased flow of positive ions into the cell. An alternative possibility is that aging causes a decrease in expression of AChRs so that there are not enough ACh receptors available to induce a change in ACh waveform. 

 To test the possibility of whether aging affects expression of AChRs, we analyzed all of the known AChRs in *Aplysia* using single neuron qPCR. Though we considered the possibility that both nicotinic and muscarinic AChRs might contribute to the observed changes in ACh responsiveness during aging, we could only analyze the eight nicotinic AChR sequences available at NCBI because the sequences of muscarinic AChRs were not found in the database. Single neuron qPCR analysis could reliably detect only five AChRs in R15 neurons. Our analysis suggests that aging causes a decrease in expression of ACh-C, ACh-E, ACh-J1 and AChR-Q, whereas AChR-G increased over two-fold during aging in R15 neurons. These results suggest that aging alters the number and composition of functional AChRs in R15. Consistent with our data that aging causes upregulation of specific channels, it was shown that aging also causes an increase in single L-type calcium channel in hippocampal neurons [60] . 

 Our gene expression results are in agreement with analysis of action potential waveform changes in response to ACh. Unlike bM-R15 neurons, ACh did not cause a change in action potential waveform in bO-R15 neurons. Herman and Gorman have shown that exposure to 4 aminopyramidine (4-AP), a blocker of voltage-dependent potassium channel, alter the shape of the action potential similar to the shape of the AP waveform we observed in response to ACh exposure in neurons collected from mature animals [61]. This suggests the possibility that voltage-gated K channels play a major role in mediating cholinergic transmission, and that aging affects the function of voltage-gated K channels in R15.

 Our electrophysiological measurements of R15 during aging discussed in this manuscript are consistent with the observation that aging is associated with a decrease in cholinergic transmission in the mammalian brain [36,37,39-43]. Furthermore we find that aging is associated with a decreased expression in specific AChRs in R15. In support of this finding, several studies have described aging-associated changes in the expression of AChRs in the mammalian brain. For example, Rogers et al reported that the neuronal nicotinic acetylcholine receptor subunit alpha 4 expression changes in the mouse brain during aging [62]. Immunostaining of this receptor was almost absent in the hippocampus in aged animals. Similarly, in aging rat brain, there was a decrease in the expression of nicotinic alpha 3-subunit mRNA expression [63,64]. These data are also supported by post mortem studies of the human brain which show that aging is associated with the reduction in levels of expression of specific ACh receptors [65-67]. The other possible mechanism that can cause a decrease in ACh-induced changes in APs is the biochemical modification of AChR such that binding of ACh to AChR is affected. In support of this possibility, Smith and Chapman [68] found that in aged rats, AChRs have a lower binding affinity, leading to poor cholinergic transmission at the neuromuscular junction. 

 Unlike other animal models such as the *Drosophila, C elegans*, and mice, *Aplysia* used in neurobiological studies are either caught in the wild or reared in the laboratory. *Aplysia* reared in the laboratory, such as those found at the NIH supported National *Aplysia* Facility at the University of Miami, are genetically more uniform and hence there are less variability between animals. Specifically by raising from specific cohort of animals under identical growth conditions such as temperature and food, individual differences among animals are minimized. However these animals are more diverse when compared to other animal models such as mouse used for aging studies. 

*Aplysia* raised in the laboratory from different egg masses show significant differences in growth characteristics. About few hundreds to thousand animals are derived from one egg mass. Within the same cohort of animals raised and maintained at the same growth conditions, *Aplysia* develop and age differently. Some become reproductively mature faster than others and exhibit significant differences in reproductive abilities, body size and weight. For example, in the cohort of animals we studied, the body size showed about a two-fold difference among animals of the same age group. 

 As shown in Figure 2, electrophysiological measurements from mature and old animals showed several different activities such as regular and irregular bursting, single spike, EPSPs and silent. Chi square analysis to detect any differences between old and mature R15 neuron did not yield any significant differences in electrophysiological activities. To understand aging associated changes, we then focused on one type of activity, regular bursting, because regular bursting is a characteristic property of R15 that several researchers have described [27-30]. About 8-10 animals out of 34 in our cohort of *Aplysia* showed regular bursting and have identified several changes during aging in bursting properties of R15. However, to obtain a comprehensive idea of aging associated changes in all the different electrophysiological activities described above, a much larger cohort of animals would be required. 

 Furthermore, analysis of aging at the level of single neurons also poses challenges. For example, there is only one R15 neuron in the animal. Because of this, one might need to start with an even larger cohort of animals because it is likely that some of the single neurons might be lost during experimental procedures. Focusing on the rhythmic bursting R15 neurons, we find several interesting differences during aging that are statistically significant. In a few instances, there were trends but they were not statistically significant. Taken together, we here report several interesting electrophysiological changes in R15 neurons during aging. Importantly, consistent with the published literature on mammalian aging that aging is associated with a decrease in response to acetylcholine [36,37,39-43], our single neuron measurements also show specific aging associated changes in response to acetylcholine. 

## Conclusion

 We have focused on regular bursting activity of R15 neurons to gain insights into electrophysiological changes associated with aging. Our measurements suggest that aging causes specific changes in synaptic transmission. There are changes in expression of ionotropic receptors, with overall changes in excitability, expression, and composition of acetylcholine receptors during the aging of R15. Our data suggests that the defects in the cholinergic system during aging are not a specialized event in mammals. However, this is a highly conserved mechanism, as we show in the case of the R15 neuron in *Aplysia* during normal aging in invertebrates. It is important to consider that R15 also showed other electrophysiological activities apart from regular bursting. An important question is to understand how changes in various electrophysiological properties correlate with specific changes in behavior during aging. This is a challenging task because R15 is implicated in multiple behaviors, and each of these behaviors might involve several other neurons with different amounts of contributions to behaviors. Mapping the circuitry consisting of specific presynaptic neurons and the follower neurons of R15 will help to understand neural correlates of behaviors and age-dependent behavioral decline. 

## Materials and Methods

### Ethics Statement

 The Institutional Biosafety Committee of The Scripps Research Institute (TSRI) has approved all of the experimental protocols (IBC Protocol 2010-019R1) described in this manuscript. There are no ethical approvals required for the research using invertebrate animals, such as the marine snail *Aplysia*. We have discussed the details of the experiments with the Institutional Animal Care and Use Committee of TSRI, and every effort was made to lessen any distress of *Aplysia*.

### Animals

 A cohort of animals (thirty-four post-metamorphic *Aplysia californica*) was maintained under standard conditions (temperature, salinity, pH, food) at the National *Aplysia* Resource Facility (University of Miami Rosenstiel School of Medicine, Florida, USA). In our study, we used animals that correspond to two age groups (6-7 months and 11-12 months old). Upon arrival in the laboratory, animals were kept in an aquarium at 16 °C, under 12:12 light-dark conditions. Animals were used for experiments within 2-3 days of arrival. 

### Preparation of ganglia

 Both mature and old animals were anaesthetized, injecting 30-35% of their body weight of a 380 mM MgCl_2_ solution, and the abdominal ganglion was then removed. We used a methodology for preparation of ganglia and ganglia electrophysiology that we recently developed (Akhmedov et al., Journal Of Visualized Experiments, accepted). Briefly, the ganglion was incubated in 0.1% dispase in 2 ml artificial seawater (ASW) at 35.5C° [ASW consisting of 450 mM NaCl, 10 mM KCl, 10 mM CaCl_2_, 55 mM MgCl_2_, 2.5 mM NaHCO_3_ and 20 mM HEPES (pH 7.4)] in order to facilitate the dissection of the connective tissue sheath overlying cells. Importantly bursting activities of R15 neurons were not affected by the protease treatment [34]. We also custom-designed a recording chamber so that the ganglion can be held in position and kept under continuous perfusion of artificial seawater. By using a polycarbonate glass-recording chamber and illuminating the chamber from the bottom, we increased the visibility of identified neurons of abdominal ganglia. The ganglion was pinned down to a Sylgard Silicone base of a bath chamber (Ø= 1 cm; vol.= 0.3ml) and constantly superfused with ASW (flow rate: 150 ul/min) at room temperature (18 ± 2 ° C), and then the sheath was dissected away and the neurons exposed to identify the R15 neuron. The R15 neuron was identified by its location, and electrical activity [29,30]. 

### R15 bioelectrical properties and activity measurements

 R15 was impaled with single intracellular sharp microelectrode (10-15 MΩ), filled with a 3M KCl solution for measuring membrane potential (voltage) and bioelectrical activity, which were recorded (10 kHz band pass) through an intracellular recording system BRAMP-01R ver. 4.2 (NPI Electronic, Tamm, Germany), digitally converted through the Instrutech ITC-18 (HEKA Instruments Inc., Bellmore, NY, U.S.A.), and then processed by Axograph X (Berkeley, CA, U.S.A.) and analyzed using a pCLAMP 10.0 (Molecular Devices Corporation, Sunnyvale, CA, USA).

 Only R15 neurons from mature and old animals with a rhythmic stable bursting activity were included in the analysis. Once R15 rhythmic bursting patterns were observed, the baseline activity was recorded for 30 minutes or more in order to check the stable activity before drug application. ACh 1 M (volvolume 0.3 uL) was directly applied into the bath using micropipette to obtain a final 1 mM concentration inside the recording chamber. Following the ACh application, R15 activity was observed for ≅ 8 minutes. Bath ACh application leads R15 to a depolarization. The depolarization rapidly reaches a plateau, indicating the point at which the drug is most effective. Any effect of ACh on the R15 membrane potential or bursting activity shown was reversible upon washing. Due to the volume of the chamber and the ASW flow rate, a complete washing is accomplished 2 minutes after ACh application. 

### Analysis of R15 membrane potential

 To study the effect of ACh on R15 membrane potential, the following parameters were analyzed: (i) membrane potential at reference condition; (ii) amplitude of the membrane potential shift (from the reference condition to the plateau); and (iii) latency of effect (time elapsed from the beginning of the shift to the plateau). To identify age-related changes in R15 membrane potential and ACh-responsiveness, the values obtained from mature animals were compared to those obtained from older animals using a Student’s t test.

### Analysis of R15 bursting activity

 In order to study ACh-related changes in the bursting activity of each recorded R15 neuron, both burst duration and the number of burst APs, before and after ACh, were separately evaluated, and the value was calculated with a duration of 90 seconds. The effect of the ACh on the basal activity was evaluated starting from the beginning of the shift of the membrane potential. Furthermore, burst duration and the number of burst APs of the first burst following neurotransmitter application were also evaluated, and the value was calculated separately. 

 The baseline values were measured during the period preceding the neurotransmitter application. For each of these analyses, we calculated the number of action potentials (before and after), and duration of each burst measured 90 seconds before and 90 seconds after ACh treatment, and averaged for one animal. Then we combined all of the data for statistical analysis. First burst action potentials and burst duration was calculated by comparing the number of action potentials in the first burst after ACh application and compared to burst immediately before ACh exposure.

### Analysis of bursting R15 action potential waveforms

 Off-line analyses of the following waveform parameters of bursts APs were studied: (i) Depolarization amplitude, (ii) Depolarization Duration, (iii) Repolarization amplitude and (iv) Repolarization duration. Baseline AP waveform parameters values were obtained from baseline bursts collected during the 90-second period preceding the neurotransmitter application. To evaluate ACh-induced changes in the bursts APs, waveform parameters were calculated from those bursts collected during a window time (with a duration of 90 seconds) starting from the beginning of the shift of the membrane potential. As in the case of measurements of bursting described above, we analyzed all of the APs produced in bursts 90 seconds before and 90 seconds after ACh treatment for each animal, and data was combined for statistical analysis (One way ANOVA followed by Tukey’s HSD post hoc test).

### Statistical analysis

 All the data shown in the figures are included in the supplementary section (Tables S1, 2, 3, 4, 5, 6 in Tables S1). For each studied parameter, data were averaged (mean ± SEM), and comparisons between old versus mature group and before and after ACh application were performed by means of Student’s t test and One Way ANOVA (GraphPad Prism, La Jolla, CA). For used statistical tests, the null hypothesis was rejected at a p value lower than 0.05. Degrees of freedom (DF) and F values are reported in the text. If not otherwise indicated, all results are expressed as mean ± SEM. Tukey’s HSD test were used for post-hoc analysis.

### Single neuron gene expression analysis

 Total RNA was isolated from *Aplysia* abdominal ganglia and single R15 neurons using the Trizol-chloroform method, and the RNA pellet was resuspended in nuclease-free water. RNA concentration and quality was measured using Nanodrop (Thermo Scientific, Waltham, MA). RNA from single neurons were subjected to two rounds of linear T7 RNA Polymerase–driven transcription using MessageAmp™ II aRNA Amplification Kit from Ambion before cDNA synthesis. cDNA was generated by reverse transcription from 1 μg of RNA from abdominal ganglia and single neurons using Quanta cDNA supermix (Quanta Biosciences) according to the manufacturer’s instructions. The expression profiles of eight AChR genes in the abdominal ganglia were first studied by qPCR using SYBR green PCR master mix (Applied Biosystems Carlsbad, CA). All of the qPCR amplifications were performed in a total volume of 10 μl containing 2 μl of H_2_O, 2 μl of cDNA, 5 μl of 2X Master Mix, 1.0 μl of 10 μM (each) forward and reverse primer (see Table S5 in Tables S1) designed based on the AChR sequences (ApAChR-C: KC411667; ApAChR-E: KC411669; ApAChR-G:KC411660; ApAChR-J1:AAL37250; ApAChR-Q:KC411665) available at NCBI (http://www.ncbi.nlm.nih.gov/). The qPCR reaction was carried out in a 7900HT Fast Real-Time PCR System (Applied Biosystems Carlsbad, CA) under the following conditions: 95°C for 10 minutes, followed by 40 cycles of 95°C for 15 s, 60°C for 1 minute. Quantification of the target transcripts was normalized to the *Aplysia* 18S rRNA reference gene using the Pfaffl method [69]. Both mature and old group had RNA samples from five animals each, and each sample was tested in four technical replicates for qPCR. One-way ANOVA was applied to identify genes with statistically significant expression levels between mature and old groups of *Aplysia*. Tukey’s multiple comparison test was used for post hoc analysis. Gene transcripts were considered differentially expressed when there was at least a 2-fold change (up or down), and the p value was set at <0.05.

## Supporting Information

Figure S1 **Resting membrane potential analysis of all the mature and old R15 neurons (bursting, irregular, silent, EPSPs, and single spike activity).** Statistical analysis suggests that resting membrane potentials of mature and old R15 neurons are not significantly different (n=12 for both mature and old, Student’s t test, p > 0.05).
(TIF)

Figure S2 **Analysis of rhythmic bursting R15 neurons.** A representative trace, baseline, ACh treatment, first burst analysis, shift and calibration graph are shown.
(TIF)

Figure S3 **Comparison of amino acid sequences of AChRs expressed in R15 neuron.**AChRs sequences obtained from NCBI were translated and the open reading frames (ORFs) were compared using multalin (http://multalin.toulouse.inra.fr/multalin/). Amino acids in red indicate identity, and blue indicate similarity among the five AChRs.
(TIF)

File S1 All supporting tables are contained File S1: **Table S1:** Intracellular measurements of R15 neurons from mature and old *Aplysia*. **Table S2:** Membrane potential measurements in old and mature R15 neurons. **Table S3:** Analysis of ACh induced changes in rhythmic bursting in mature and old R15 neurons. **Table S4:** Single spike analysis of bursting. Measurements of amplitude and duration of depolarization and repolarization of AP waveform. **Table S5:** Sequences of qPCR primers used in the gene expression analysis. **Table S6:** AChR gene expression data obtained from single R15 neuron qPCR experiments.
(XLSX)
