# Supplementary figures and images for: Decreased Response to Acetylcholine during Aging of Aplysia Neuron R15
Source: PLoS One. 2013 Dec 27;8(12):e84793. doi: 10.1371/journal.pone.0084793 (PMC3874043; doi:10.1371/journal.pone.0084793)

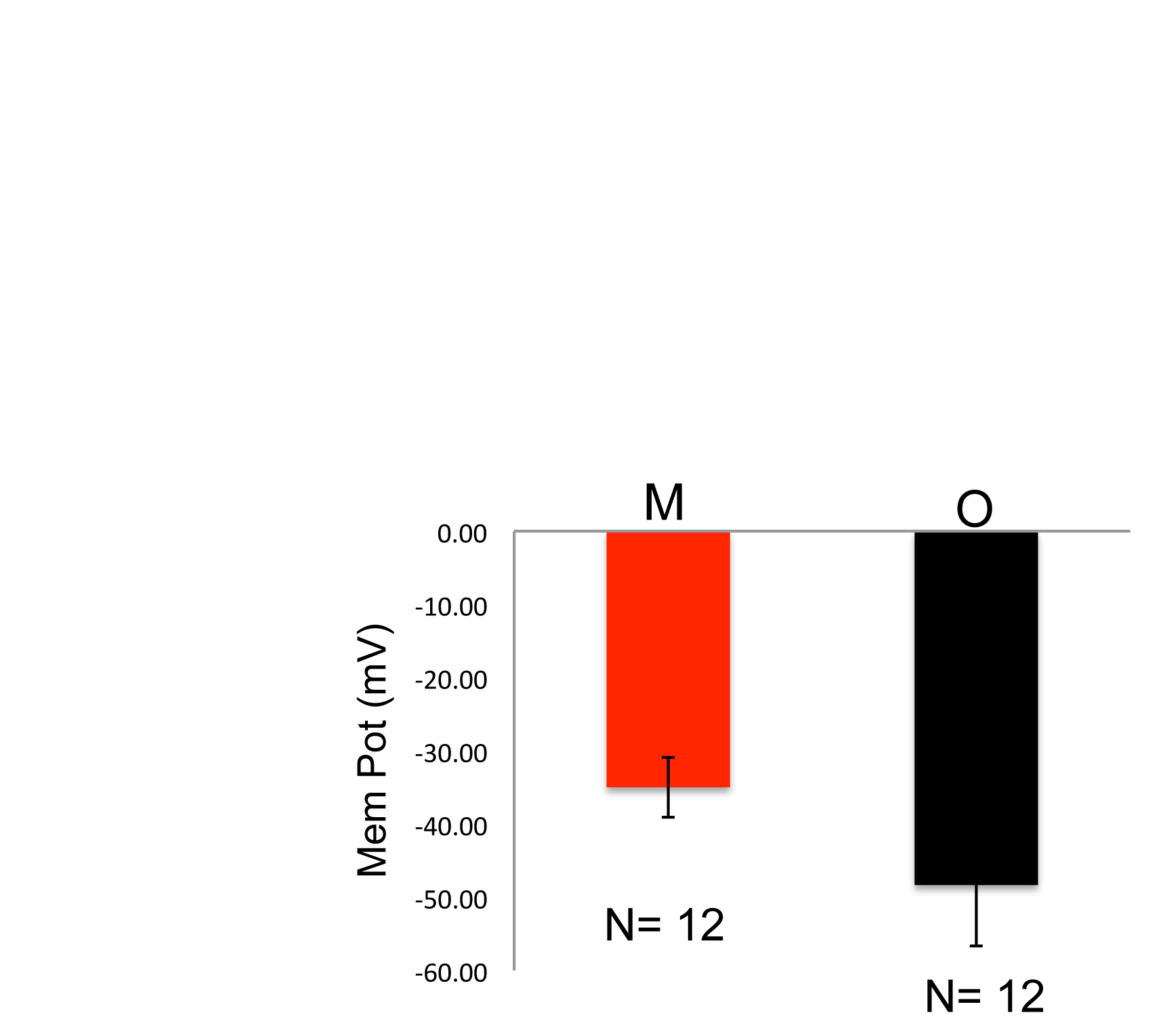

Supplement: Figure S1 — Resting membrane potential analysis of all the mature and old R15 neurons (bursting, irregular, silent, EPSPs, and single spike activity). Statistical analysis suggests that resting membrane potentials of mature and old R15 neurons are not significantly different (n=12 for both mature and old, Student’s t test, p > 0.05). (TIF) [file pone.0084793.s001.tif]

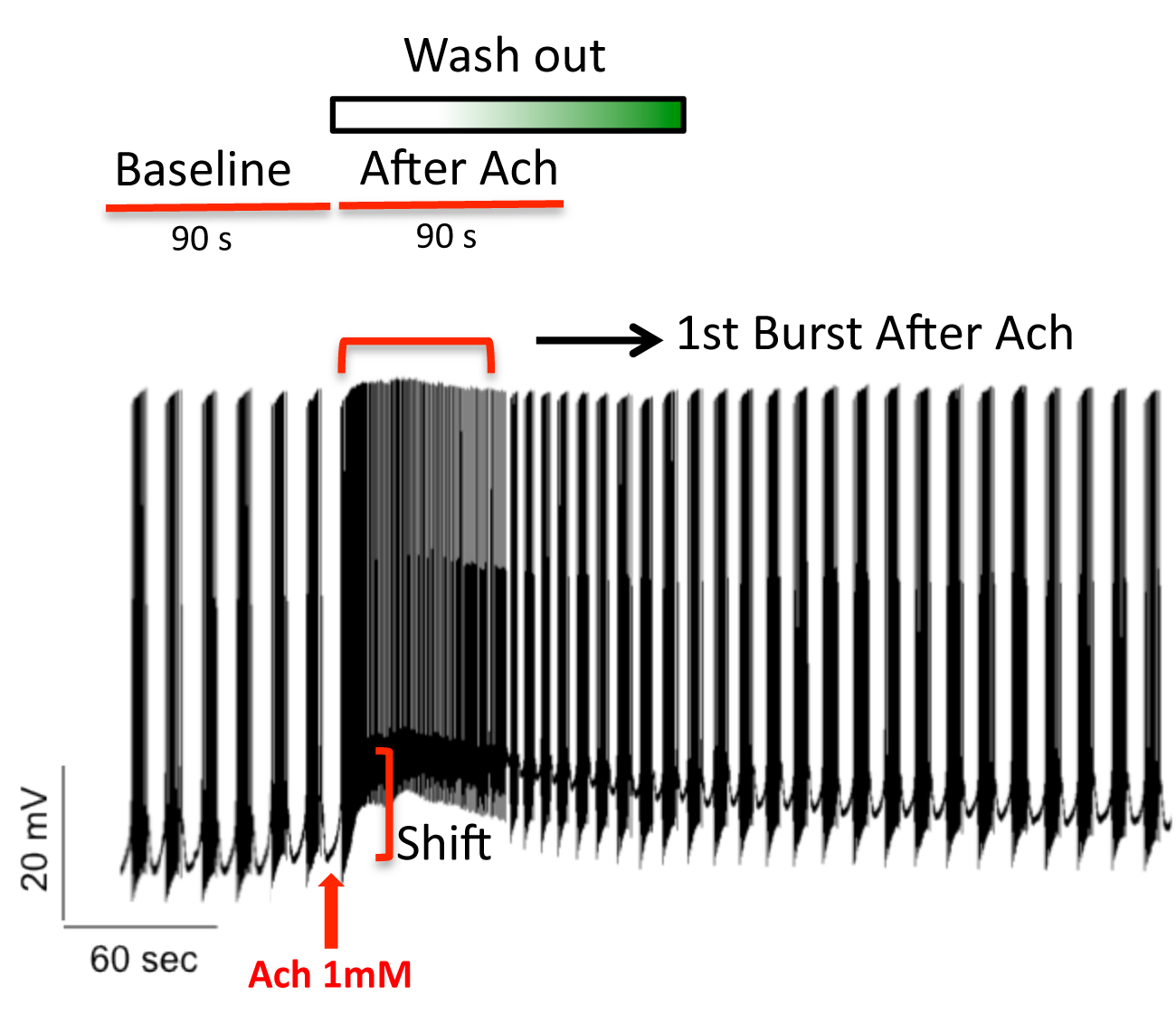

Supplement: Figure S2 — Analysis of rhythmic bursting R15 neurons. A representative trace, baseline, ACh treatment, first burst analysis, shift and calibration graph are shown. (TIF) [file pone.0084793.s002.tif]

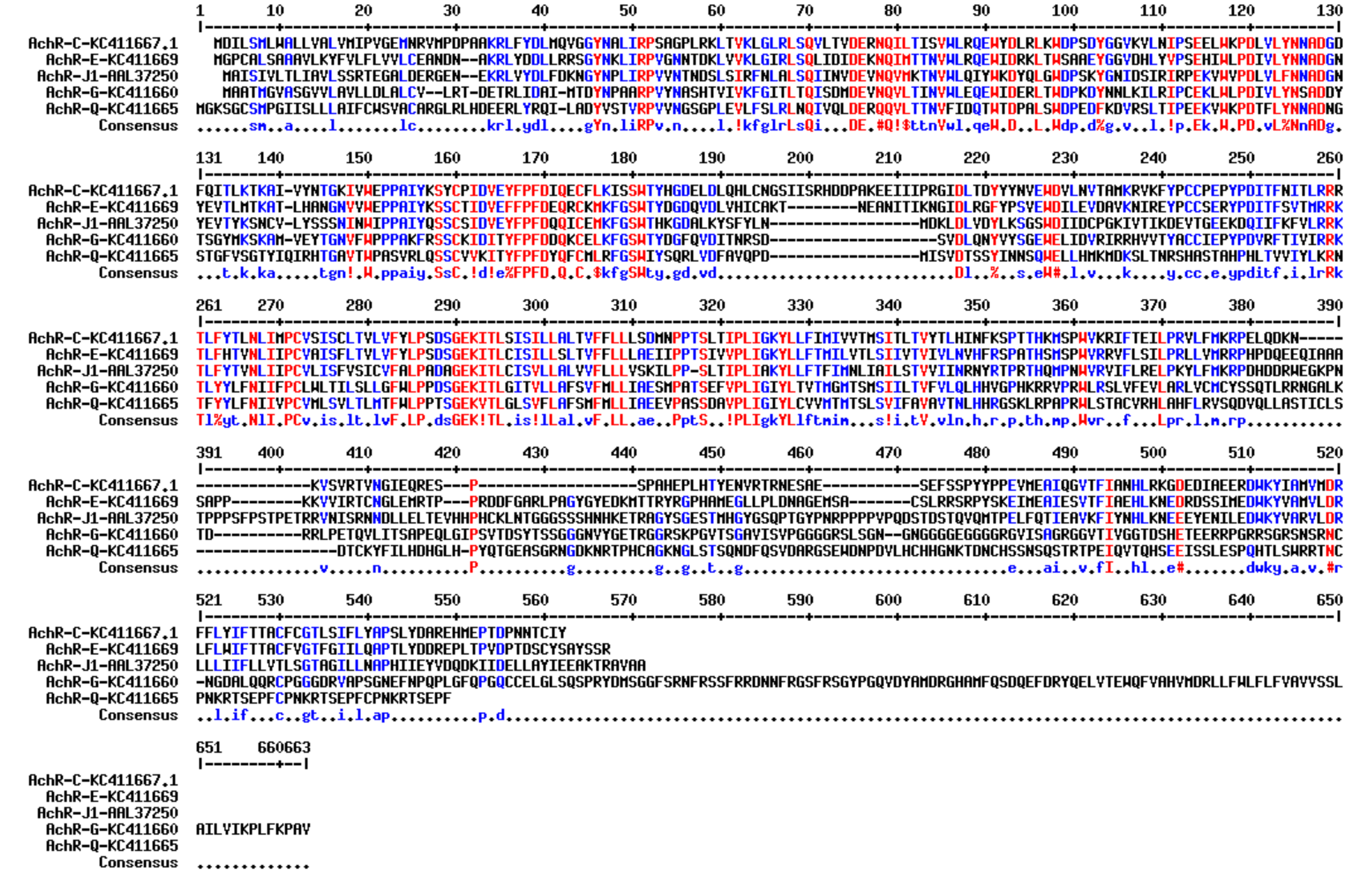

Supplement: Figure S3 — Comparison of amino acid sequences of AChRs expressed in R15 neuron.AChRs sequences obtained from NCBI were translated and the open reading frames (ORFs) were compared using multalin (http://multalin.toulouse.inra.fr/multalin/). Amino acids in red indicate identity, and blue indicate similarity among the five AChRs. (TIF) [file pone.0084793.s003.tif]
